# DNA Methylation and the Evolution of Developmental Complexity in Plants

**DOI:** 10.3389/fpls.2018.01447

**Published:** 2018-10-04

**Authors:** Katharina Bräutigam, Quentin Cronk

**Affiliations:** ^1^Department of Biology, University of Toronto Mississauga, Mississauga, ON, Canada; ^2^Department of Botany, The University of British Columbia, Vancouver, BC, Canada

**Keywords:** methylome, epialleles, plant evodevo, epigenotype, epi-evodevo, histones, chromatin, Baldwin effect

## Abstract

All land plants so far examined use DNA methylation to silence transposons (TEs). DNA methylation therefore appears to have been co-opted in evolution from an original function in TE management to a developmental function (gene regulation) in both phenotypic plasticity and in normal development. The significance of DNA methylation to the evolution of developmental complexity in plants lies in its role in the management of developmental pathways. As such it is more important in fine tuning the presence, absence, and placement of organs rather than having a central role in the evolution of new organs. Nevertheless, its importance should not be underestimated as it contributes considerably to the range of phenotypic expression and complexity available to plants: the subject of the emerging field of epi-evodevo. Furthermore, changes in DNA methylation can function as a “soft” mutation that may be important in the early stages of major evolutionary novelty.

## Introduction – Epigenetics and Development

The evolution of complex organs and systems such as vasculature, rooting structures, flowers, or seeds is key to the success of land plants. Concomitantly, we observe an increase in the number of different major cell types that can range from 12 or 13 in liverworts and hornworts to 44 in flowering plants (Niklas et al., 2014). An increase in distinct cell types requires a precise and increasingly complex interpretation of the genome to initiate differentiation and maintain cell identity across mitotic divisions thus allowing for tissue and organ formation. In addition to a diversity in organ form, sessile land plants can exhibit an impressive phenotypic plasticity that contributes to their ability to colonize, grow, and reproduce in unpredictable terrestrial environments. As opposed to vertebrates with a fixed body plan, land plants are modular in construction, and individuals of the same genotype can vary in size, placement, and frequency of organs (such as leaves) depending on the environment they are exposed to. Thus, there is a complex interplay between internal and external signals. At the molecular level, land plants have a remarkable diversity of epigenetic pathways at their disposal that likely play key roles in developmental complexity, phenotypic diversity, and adaptive capacity (Bossdorf et al., 2008; Bräutigam et al., 2013; Kooke et al., 2015).

Covalent modifications of DNA and histones, together with histone variants, chromatin modulating factors, and non-coding RNAs shape the epigenetic landscape that controls development in many eukaryotes, particularly complex eukaryotes. However, it should be noted that there are general differences between animal and plant development that may affect the use of epigenetic mechanisms in gene regulation. One such difference is the relative importance of cell lineage in development. In many animals there is sustained maintenance of cell identity in lineages, spanning many mitoses. In *Caenorhabditis elegans* for instance “rigidly determined cell lineages generate a fixed number of progeny cells of strictly specified fates” (Sulston and Horvitz, 1977). In plants, cell fate is more likely to be determined by cell-context signaling, i.e., cell position relative to its neighbors (Pennell et al., 1995). This may help to explain some plant-animal epigenetic differences. For instance, it has been suggested that gene repression by the chromatin remodeling Polycomb-group (PcG) proteins is generally less long lasting and more responsive to developmental and environmental cues in plants than it is in animals (Köhler and Grossniklaus, 2002). This in turn is perhaps related to kingdom-specific diversification of PcG proteins. An absence in plants of certain animal-specific PcG proteins that are required for long-term maintenance of gene repression in animal cell lineages is observed along with duplications of other PcG components (Köhler and Grossniklaus, 2002).

Similarly, plants differ from animals in that the plant “germline” (such as it is) is set at a late stage mostly by environmental or cell-context signaling (Whipple, 2012) whereas in animals the germline is generally determined early and is lineage based. There is, however, evidence emerging for a reduced number of stem cell divisions in axillary meristems, resulting from early set-aside, analogous to the animal germline (Burian et al., 2016). In plants the situation is complicated by the intercalation of a gametophyte generation between meiosis and gametogenesis, but in angiosperms the germline is interpreted as originating in the archesporial (meiotic fate) cells of anther or ovule (Kelliher and Walbot, 2012). The lack of separation between germ line and soma in plants may make transgenerational transmission of epialleles intrinsically more likely in plants (Jablonka and Lamb, 1998; Cronk, 2001) as the setting of the animal germline involves extensive methylation reprogramming (Lees-Murdock and Walsh, 2008). Epigenetic reprogramming in the germline of angiosperms also occurs, but it is highly specific and complex (Slotkin et al., 2009) and, unlike in animals, methylation in CG and CHG contexts is largely retained (Calarco et al., 2012).

Land plants (embryophytes) have a further major distinction from animals in that they have an alternation of haploid and diploid generations. In this, the same set of genes (in diploid vs. haploid form) generates two morphologically divergent organisms. It has been suggested that epigenetic reprogramming is likely to be involved in this extraordinary phenomenon (Cronk, 2001) and there are now studies that bear this out (Mosquna et al., 2009; Okano et al., 2009) and some that implicate DNA methylation as part of this control (Yaari et al., 2015).

In a broad evolutionary context, a particularly interesting taxonomic group are streptophyte algae (Zygnematophyceae, Coleochaetophyceae, Charophyceae, and Klebsormidiophyceae), as they comprise the lineage in which the embryophytes evolved. It is therefore of especial interest that the *Klebsormidium* and *Chara* genomes have recently been sequenced (Hori et al., 2014; Nishiyama et al., 2018), as this opens the way to future studies of methylation in these taxa. The closest algal group to the land plants is the Zygnematophyceae (Turmel et al., 2006; Timme et al., 2012; Ruhfel et al., 2014; Wickett et al., 2014; Gitzendanner et al., 2018), and further sequencing in this group will also assist the elucidation of DNA methylation processes directly ancestral to those of the embryophytes.

The evolution of epigenetic function as it affects the evolution of plant development (“epi-evodevo”) will be a fertile field of enquiry. DNA methylation is one of the better-known epigenetic mechanisms, yet determining how the evolving methylome is linked to the evolution of plant form is still a major challenge.

## Constitutive and Facultative Epigenetic Control of Development

In plants, developmental control through epigenetic mechanisms can be considered either constitutive (internal signals) or facultative (external signals). Constitutive developmental control is based on internal developmental cues and is characteristic of organism-specific normal development. In contrast, facultative developmental control is based on external environmental cues and is how organisms (especially plants) respond to different environments by developmental plasticity giving rise to environment-specific phenotypes.

### Epigenetic Control in Constitutive Development

Well-documented examples for constitutive epigenetic control in development relate to events central to reproduction and seed development in angiosperms/*Arabidopsis*. If disrupted, these can have detrimental effects on the formation of reproductive tissues and seeds (Chaudhury et al., 1997). Such examples include global demethylation of the genome in vegetative companion cells in the female and male gametophyte to reinforce TE silencing in the sperm and egg cells as well as the embryo (Hsieh et al., 2009; Slotkin et al., 2009; Law and Jacobsen, 2010), genomic imprinting in the nourishing tissue, the endosperm (Rodrigues and Zilberman, 2015), or extensive DNA methylation changes during seed development (Kawakatsu et al., 2017).

Double fertilization to form a triploid nutritive tissue (endosperm) is an angiosperm innovation of enormous evolutionary importance, and one that requires epigenetic controls during development. Two sperm cells are involved: one sperm nucleus fertilizes the egg to form the zygote and ultimately the embryo while the other sperm nucleus fuses with the two nuclei of the central cell to give rise to the endosperm, i.e., the tissue that nourishes the developing embryo. Intriguingly, global de-methylation is observed in the central cell of the female gametophyte prior to fertilization. This results in a marked increase of small interfering RNAs (siRNAs) likely due to the re-activation of transposons. These siRNAs are thought to migrate to the egg cell and reinforce TE silencing in the egg cell and likely the developing embryo (Hsieh et al., 2009; Law and Jacobsen, 2010). Similar demethylation and reinforcement events have also been observed in the male gametophyte (Slotkin et al., 2009).

Due to the demethylation in the central cell, maternal and paternal genomes in the endosperm differ in their DNA methylation levels in the endosperm. This can result in the parent-of-origin dependent gene expression (genomic imprinting) which affects a number of genes including the FERTILIZATION INDEPENDENT SEEDS (FIS)-complex genes *MEDEA* and *FIS2*. Misexpression of *MEDEA*, for example, can result in seed abortion and/or abnormal embryo development (Grossniklaus et al., 1998). Theories for the emergence of genomic imprinting in plants include parental conflicts in resource allocation, co-adaptation of maternal and embryonic characteristics or dosage-dependent gene regulation, however, much remains to be learned about the biological significance and role in plant evolution (Rodrigues and Zilberman, 2015). Furthermore, following fertilization and initial embryo development, genome-wide changes in DNA methylation shape the epigenome during seed development, dormancy, and germination. During seed development, extensive hypermethylation especially in CHH context in TEs is observed which is reset during germination (Kawakatsu et al., 2017).

Another example of constitutive is provided by the role of CURLY LEAF (CLF) in normal leaf development (Goodrich et al., 1997). CLF is a polycomb group (PcG) protein, a group that functions by remodeling chromatin to maintain stable gene repression through many mitoses, i.e., marking a cell lineage. PcG proteins form modular multimeric complexes that fulfill diverse roles in development. Several genes coding for polycomb repressive complex 2 (PRC2) components have diversified in plants. The EMBRYONIC FLOWER 2-containing complex, for example, maintains vegetative development and represses reproduction while the FIS-complex prevents seed development in the absence of fertilization in *Arabidopsis* (Hennig and Derkacheva, 2009).

### Epigenetic Control in Facultative Development

An example of facultative developmental control is the vernalization response. This is the plant’s memory of having passed through winter, giving it the competence to flower. It involves the PcG protein VERNALIZATION 2 (VRN2) (Wood et al., 2006) along with a complex suite of epigenetic pathways including chromatin remodeling factors, histone modifications, non-coding RNAs, and DNA methylation (He, 2012). Vernalization is a facultative epigenetic response whereby environmental cues determine the developmental outcome between two phenotypic states, vegetative and flowering.

Similarly, perception of the light environment during fundamental transitions in development such as germination and photomorphogenesis (i.e., the transition to autotrophic growth in the seedling) relies on environmental signals that are translated into altered chromatin states and consequently massive transcriptional reprogramming. Light exposure perceived by photoreceptors eventually leads to reduced hypocotyl elongation, the opening of embryonic leaves (cotyledons), and chlorophyll biosynthesis. This process involves major changes in the genome organization to induce permissive chromatin states at hundreds of light-inducible genes. Such changes encompass an increase in size of the nucleus, a moderate ploidy level increase through endoreduplication, heterochromatin condensation, and histone modifications (Bourbousse et al., 2015).

Environmental signals can be integrated into the chromatin landscape in such a way that development and genome function are fine-tuned. Morphological changes, often subtle, can be observed in postembryonic development in response to variable environmental conditions and challenges. Environmental signals can be incorporated via hormone signaling into the chromatin of vegetative meristems. This in turn can modulate root architecture or leaf development (Xiao et al., 2017). A good example of a morphological response to environmental stress is the increased leaf trichome formation in the yellow monkey flower *Mimulus guttatus* as defense against insect herbivory. Trichomes are epidermal outgrowths and a model for cell differentiation and patterning. In *M. guttatus*, trichome density is not only stimulated in herbivory exposed plants but this trait is also epigenetically transmitted to their non-stressed offspring, and is likely mediated by changes in the expression of a MYB transcription factor (Scoville et al., 2011).

A further example is the ability of *Arabidopsis* plants to fine-tune water relations epigenetically. Low relative humidity induces hypermethylation at *SPEECHLESS* (*SPCH*), a gene in the stomatal developmental pathway. This correlates with reduced *SPCH* gene expression and a reduced stomatal index (Tricker et al., 2012). Intriguingly, DNA methylation pattern at *SPCH* and stomatal phenotype were transmitted to progeny, although both were reversable under repeated stress treatment in these progeny (Tricker et al., 2013a,b).

Finally, epigenetic recombinant inbred lines (epiRILs) that are isogenic but differ in their DNA profiles are useful tools to study the effects or epigenetic variation on plant phenotype. Work in *Arabidopsis* epiRILs shows that the variation in morphological and physiological traits (e.g., flowering time or plant height) among epiRILs is comparable to that observed among natural accessions highlighting the potential of DNA methylation variation in modulating phenotype (Johannes et al., 2009; Roux et al., 2011).

Thus, DNA methylation and other modifiers of the chromatin landscape can effectively shape plant phenotype without, or prior to, genetic change; i.e., they can be considered a “conditions-sensitive ability to create diversity […] related to both ontogenetic adaptive plasticity and evolutionary adaptation” (Jablonka, 2013).

## DNA Methylation in Green Plants

**FIGURE 1:**
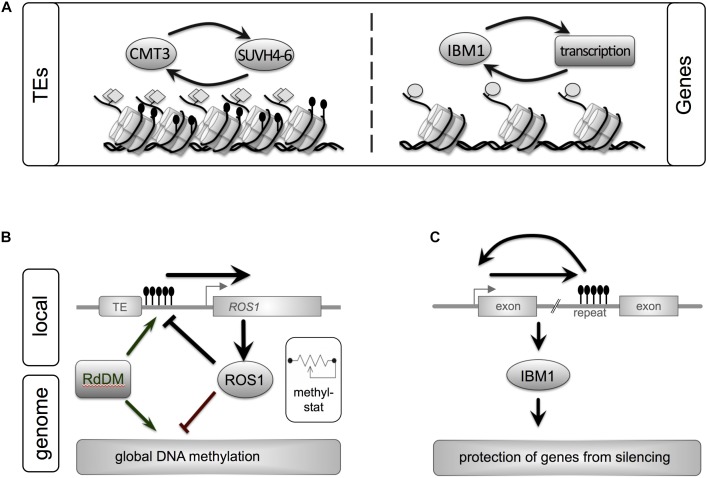
Epigenetic maintenance of genome integrity involves both repeats and genes in plant genomes. A close interplay between genetic elements and epigenetic pathways ensures homeostasis of eu- and heterochromatin. **(A)** Silent transposons (TEs) are characterized by heterochromatic marks such as DNA methylation (black lollipops, non-CG context) and histone methylation (H3K9me2, gray diamonds). These marks are maintained by a self-reinforcing loop between CMT3, a DNA methyltransferase, and histone methyltransferases. Another feedback loop stabilizes active genes. IBM1, a histone demethylase prevents repressive H3K9me2 histone mark from spreading into genes. **(B)** ROS1, a central enzyme that removed DNA methylation marks, links local DNA methylation within its own promoter to genome-wide DNA methylation levels: acting as “methylstat.” Methylation of a sequence with the promoter of ROS1 close to a TE promotes expression of ROS. ROS1 in turn demethylates its own promoter and other regions in the genome. By monitoring the genome’s methylation status and adjusting its own expression ROS1 contributes to fine-tuning of the cell’s DNA methylation levels. Active DNA methylation is performed by RdDM or Met1. **(C)** The histone demethylase IBM1 contains a heterochromatic repeat in one of its introns. Correct IBM1 expression is crucial for the protection of genes from heterochromatic marks (H3K9me2, non-CG methylation) and thus correct genome-wide expression profiles. Reduction of DNA methylation at this repeat reduces IBM1, which leads to genome-wide hypermethylation in thousands of genes and a number of developmental defect. Gene and regulatory loops shown reflect processes in Arabidopsis (Lei et al., 2014; Sigman and Slotkin, 2016).

Methylation of cytosine (5-methylcytosine) is a common covalent modification of DNA which can be passed on across mitotic and meiotic cell divisions. While it does not alter the primary sequence of the DNA and thus the genetic information, DNA methylation plays important roles in maintenance and regulation of genome structure and function (**Figure 1**). For example, it contributes to the organization of chromatin into condensed heterochromatic regions, is involved in repeat silencing, and has been implicated in the regulation of gene expression and recombination. It can affect central biological processes ranging from normal cell function to genomic imprinting, regulation of development or responses to environmental cues, and is of relevance in heterosis (hybrid vigor) or polyploidization events.

In addition to 5-methylcytosine discussed herein, plant DNA can contain a number of non-canonical base modifications at low frequencies. These including various oxidized derivatives of 5-methylcytosine or *N*^6^-methyladenine (6mA) (Liu et al., 2013; Zhou et al., 2018). While such base modifications have been rarely studied in a plant evolutionary context, 6mA might emerge as an interesting epigenetic mark in plant and animal systems (Greer et al., 2015; Zhang et al., 2015; Zhou et al., 2018).

For DNA methylation, the sequence context is of relevance. Whereas animals (metazoa) are characterized predominantly by CG methylation, DNA methylation in plants occurs in all sequence contexts: symmetric CG, CHG, and asymmetric CHH (H = A, T, or C) which are set and maintained by context-specific but partially overlapping molecular pathways. A number of distinct DNA methyltransferases both generate (*de novo*), and subsequently maintain, DNA methylation at three sequence contexts: MET1 maintains CG methylation, plant-specific CHROMOMETHYLASES (CMTs) pathways target CHH (CMT2) and CHG sites (CMT3 and CMT2) in repeats and transposons, and asymmetric CHH methylation is maintained via DRM2 through persistent *de novo* methylation (RNA-directed DNA methylation pathway, RdDM). Names here refer to proteins in *Arabidopsis* (Law and Jacobsen, 2010; Stroud et al., 2013).

DNA methylation is an ancient epigenetic mark. In the green lineage (Viridiplantae), it is found in all major taxonomic groups including Chlorophycean green algae, liverworts, mosses, ferns, gymnosperms, or angiosperms (Feng et al., 2010; Zemach et al., 2010; Takuno et al., 2016). Given its function and conservation, epigenetic regulation via DNA methylation is likely an important factor in plant evolution. The variability of methylomes among taxonomically diverse plants has recently attracted increasing attention (Niederhuth et al., 2016; Vidalis et al., 2016). However, only recently are we beginning to understand how DNA methylation patterns are shaped over evolutionary time scales, and how individual epigenetic variability contributes to phenotypic variation and adaptive potential (Bossdorf et al., 2008; Bräutigam et al., 2013; Vidalis et al., 2016). Here we bring together the current understanding of DNA methylation in plant evolution and development, drawing widely on studies from across the green plants.

## DNA Methylation of Transposable Elements

Within all plant genomes, DNA methylation shows a non-random distribution: DNA methylation is universally enriched in repetitive regions such as transposable elements (TEs), centromeric repeats, and rDNA (Slotkin and Martienssen, 2007; Feng et al., 2010; Zemach and Zilberman, 2010). Active TEs are mutagenic and can disrupt genes, regulatory regions, and affect genome integrity. Most existing TEs are, however, inactive, i.e., silenced and/or non-functional. Silencing of TEs has been proposed as one of the original functions of DNA methylation pathways (Slotkin and Martienssen, 2007; Mirouze and Vitte, 2014; Sigman and Slotkin, 2016).

Transposable elements show increased levels in DNA methylation in all sequence contexts (most prominently CG and CHG), a feature detected in almost all of the studied plant epigenomes ranging from the moss *Physcomitrella* to gymnosperms and angiosperms (Chan et al., 2008; Feng et al., 2010; Zemach et al., 2010; Niederhuth et al., 2016; Lang et al., 2018). Preferential methylation of repeats has also been reported in the distantly related green algae *Chlamydomonas*, *Chlorella*, and *Volvox* (Feng et al., 2010; Zemach and Zilberman, 2010). Work in the model plant *Arabidopsis thaliana* has shown that silent TEs adopt a distinct chromatin state, characterized by the repressive histone H3 lysine 9 dimethylation (H3K9me2) mark in combination with elevated DNA methylation levels and other histone modifications (Cokus et al., 2008; Roudier et al., 2011). This repressive heterochromatin of silent TEs is one of four major chromatin states described for the *A. thaliana* genome, and distinct from chromatin of actively transcribed genes, polycomb-repressed genes, and intergenic regions (Roudier et al., 2011).

Transposable element repression is mediated by overlapping mechanisms (double lock) including a re-inforcement loop between H3K9me2 and (non-CG) DNA methylation, and it also involves small interfering RNA (siRNA) (Slotkin and Martienssen, 2007; Roudier et al., 2011; Mirouze and Vitte, 2014; Sigman and Slotkin, 2016). While the boundaries between heterochromatic TEs and euchromatic genes are generally reinforced, heterochromatin can sometimes spread from silenced TEs and influence the expression of genes in their vicinity. Examples in *A. thalian*a include *FLOWERING WAGENINGEN (FWA)* or *BONSAI (BNS*) (Soppe et al., 2000; Saze and Kakutani, 2007). In addition, there are multiple further mechanisms by which TEs can influence the expression of genes in *cis* and in *trans* as reviewed previously (Slotkin and Martienssen, 2007; Mirouze and Vitte, 2014; Sigman and Slotkin, 2016).

## From Control of TEs to Control of Genes: The Methylation Transition

**FIGURE 2:**
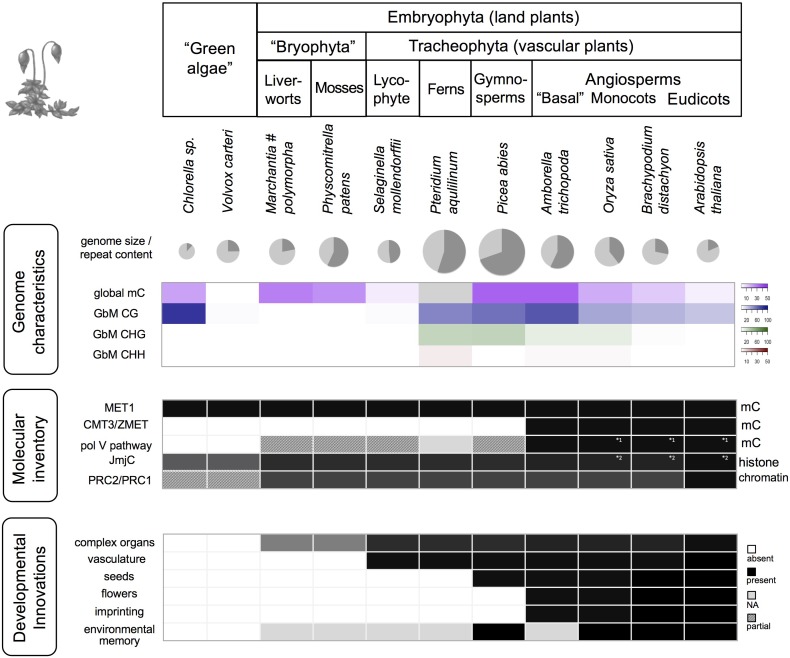
Genomic, epigenomic, and morphological characteristics in Viridiplantae. The various taxonomic groups of the Viridiplantae are characterized by differences in global genome characteristics, the complement of epigenetic factors (selected factors/pathways shown), and plant function. Genome size is visualized for selected species (diameter corresponds to log (genome size)), along with the proportion of repeats (dark gray slice), global DNA methylation (mC), and gene body methylation (GbM). Dark cells indicate presence, white cells absence, light gray: missing data, shaded: pathway/complex likely incomplete due to missing components, or due to factors that differ in protein size or lack a characteristic domain. Data are based on Hennig and Derkacheva (2009); Feng et al. (2010), Zemach and Zilberman (2010); Raj et al. (2011), Matzke et al. (2015); Takuno et al. (2016) and Bewick et al. (2017). MET1, DNA METHYLTRANSFERASE1; CMT3, CHROMOMETHYLASE 3; pol V, RNA polymerase V; JmjC, JumonjiC domain-containing histone demethylases; PRC, Polycomb group repressive complex. Note (#): While mostly low, GbM can be prominent in particular stages of *M. polymorpha* life cycle, especially in antherozoids (male gametes) (Schmid et al., 2018).

**FIGURE 3:**
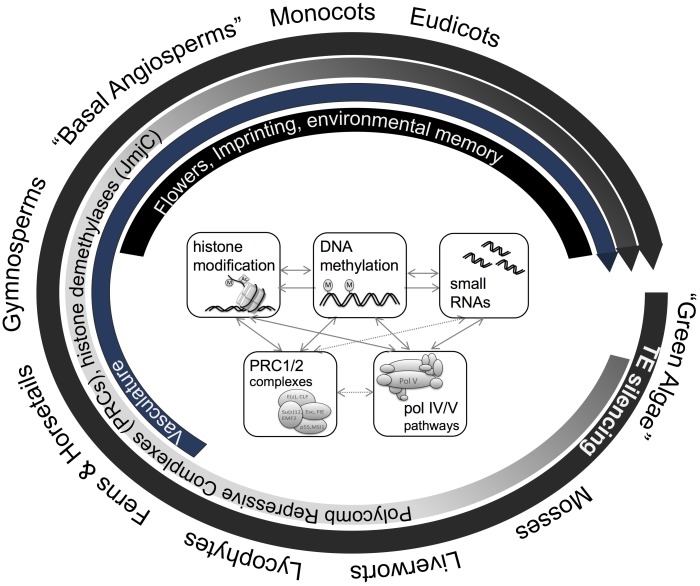
Macro-epi-evodevo. The evolution of land plants is characterized by various developmental innovations, which are accompanied by emergence, co-option, and divergence of various genome- and epigenome-related processes. Plants uniformly employ DNA methylation and related pathways for Transposon (TE) silencing, genomes of ferns and more evolved plants show gene body methylation, and histone modifying enzymes (JmjC histone demethylases) as well as chromatin modifying complexes (PRC1/2) have diversified during the evolution of land plants. More recently, new pathways such as CMT3 or pol V pathways have emerged during the evolution of angiosperms which are involved in DNA methylation in non-symmetric sequence context or have been hypothesized to play a role in genome-reduction after polyploidization. In parallel, new phenomena such a genomic imprinting, complex organ development, and environmental memory occur in angiosperms.

The use of DNA methylation to control TEs is nearly ubiquitous in eukaryotes and is thus apparently an ancient feature. The additional molecular inventory to use DNA methylation to control genes, by the specific methylation of promoters and transcriptional start sites, appears to have arisen later in evolution but is apparently ubiquitous in the embryophytes (**Figures 2, 3**).

The moss, *Physcomitrella patens* has a DNA METHYLTRANSFERASE 1 (MET1) homolog as well as a CHROMOMETHYLASE (CMT) gene, RdDM methylases and two additional DNA methyltransferases (Malik et al., 2012). Loss of these genes results in overexpression of other genes in the moss genome implying they that they have a repressive role in gene transcriptional control.

Given the extensive level of transcriptional control by DNA methylation evident in land plants, it would be fair to ascribe considerable developmental significance to DNA methylation. It would follow that the evolution of gene expression control from TE control is one of the most significant evolutionary transitions in the emergence of complex organisms. In fact, loss of key genes, such as *MET1*, can have rather variable impacts on development. In the moss *Physcomitrella*, for instance, plants lacking the *MET1* homolog failed to produce sporophytes (Yaari et al., 2015). This drastic effect has been ascribed to impaired gamete development, fertilization or early steps in embryo development due to concomitant loss of CG methylation. In contrast, loss of the *MET1* homolog, had, surprisingly, no effect on gametophyte development (Yaari et al., 2015); however, treatment of the gametophyte with the methyltransferase inhibitor zebularine does produce abnormal phenotypes (Malik et al., 2012). These observations in *Physcomitrella* indicate strong life-cycle specificity of DNA methylation effects as well as partial redundancy in the DNA methylation machinery (Malik et al., 2012; Yaari et al., 2015).

Similarly, *Arabidopsis* mutants with altered DNA methylation levels show diverse phenotypic characteristics. Lack of functional AtMET1 which results in strongly reduced CG methylation, leads to a number of abnormalities including small plant size, reduced fertility, changes in flowering time, or altered floral morphologies (Finnegan et al., 1996; Kankel et al., 2003; Mathieu et al., 2007). Here, the late flowering phenotype can be attributed to ectopic *FWA* expression due to DNA methylation loss at a TE upstream of the gene (Soppe et al., 2000). Moreover, loss of MET1 in *Arabidopsis* results in impaired development in a significant proportion of embryos (Xiao et al., 2006). These embryos show misregulation of gene expression, abnormal patterns of cell division (planes and number or cell divisions) or improperly formed auxin gradients (Xiao et al., 2006). While some *Arabidopsis* mutants with lesions in individual components of the DNA methylation machinery such as the DNA methyltransferases CMT3, DRM1, or DRM2 grow like wildtype plants, simultaneous loss of multiple components results in developmental defects (e.g., *drm1 drm2 cmt3* triple mutant with loss of non-CG methylation (Cao and Jacobsen, 2002; Chan et al., 2006), or enhances phenotypic abnormalities: *met1 cmt3* double mutant (Xiao et al., 2006).

The mild defects observed in several individual *Arabidopsis* mutants thus likely reflect redundancies of DNA methylation pathways and the generally low DNA methylation levels in this plant (**Figure 2**). Plant species with larger genomes, higher repeat content, and global DNA methylation levels such as rice or maize show more severe phenotypes or lethality in DNA methylation mutants (Erhard et al., 2009; Hu et al., 2014). For example, lack of OsMET1 in rice resulted in abnormal seeds and seedling lethality (Hu et al., 2014), and in contrast to *Arabidopsis*, rice chromomethylase (*cmt3a*) mutants produced less biomass, showed low fertility and were characterized by complex expression changes of cellular genes and mobilization of TEs (Cheng et al., 2015). These examples show that while DNA methylation retains a central function in TE management, it also plays key roles in the regulation of developmentally important genes, likely as part of a complex regulatory network, and with built in redundancies.

## Diversification of DNA Methylation Pathways

While DNA methyltransferase 1 (MET1), CMT, and RdDM functionality is present in all land plants (i.e., setup and maintenance of DNA methylation in all sequence contexts), there is evident evolutionary diversification in several DNA methylation pathways, indicating an expansion of their roles. For example, the complex pol V branch of RdDM pathway (**Figure 2**) is only fully functional in angiosperms and has been hypothesized to play a role in diploidization and genome reduction after whole-genome duplication shock (Matzke et al., 2015). Similarly, CMT3, involved in the maintenance of non-CG methylation through a reinforcement loop with histone methylation (H3K9me2) is angiosperm-specific and can be counteracted by a angiosperm-specific histone demethylase (Increase in *BONSAI* Methylation 1, IBM1) that prevents spreading of DNA methylation. More generally, histone demethylases and PRC components have diversified gradually during land plant evolution (shown for JmjC type in **Figure 2**) potentially contributing to the regulation of increased developmental complexity and extensive interactions with the environment. **Table 1** gives some examples of the control of developmental pathways by targeted methylation or demethylation.

**Table 1:** Some examples of developmental genes regulated by, or affected by, methylation status.

Gene	Developmental pathway	Notes	Reference
*AGAMOUS* (*AG*)/*SUPERMAN* (*SUP*)	Floral whorls	Mutants with hypermethylation in *AG* and *SUP* gene bodies display floral abnormalities	Jacobsen et al., 2000
*APOLO* lncRNA	Auxin	Auxin triggers demethylation of *APOLO* DNA and lncRNA production	Ariel et al., 2014
*CYCLOIDEA* (*CYC*)	Floral zygomorphy	Naturally occurring hypermethylated teratomorph of *Linaria* abolishes *CYC* expression and results in change of floral symmetry	Cubas et al., 1999
*EPIDERMAL PATTERNING FACTOR 2* (*EPF2*)	Stomatal development	Normal development requires demethylation by *ROS1*	Yamamuro et al., 2014
*SPEECHLESS (SPCH)*	Stomatal patterning (stomatal index)	Low relative humidity induces methylation at *SPCH*	Tricker et al., 2012
*FLOWERING WAGENINGEN* (*FWA*)	Flowering time	Epialleles of *FWA* confer late flowering phenotype	Soppe et al., 2000; Kinoshita et al., 2004
*MEDEA* (*MEA*)	Seed development	Allele-specific demethylation by *DEMETER* (*DME*) required for the establishment of self-imprinting by *MEDEA*	Gehring et al., 2006
*PHABULOSA* (*PHB*)/*PHAVOLUTA* (*PHV*)	Leaf development; polarity	*PHB* and *PHV* coding sequences have functionally important methylation downstream of microRNA binding site	Bao et al., 2004
*RIPENING INHIBITOR* (*RIN*)/*NON-RIPENING* (*NOR*)	Fruit ripening in tomato	Demethylation of ripening genes, including *RIN* and *NOR*, by the tomato homolog of *ROS1*, is required for normal ripening	Lang et al., 2017


*All genes are from Arabidopsis except CYC (Linaria) and RIN/NOR (tomato).*

Further complexity in DNA methylation pathways result from linking a repeat sequence to the plant’s methylation status. An interesting example of this, for the control and fine-tuning of gene expression and development in *Arabidopsis*, is provided by *ROS1* (*REPRESSOR OF SILENCING1*) and *IBM1* (**Figures 1B,C**). Both can act as epigenetic rheostat or “methylstat” to establish a genome-specific DNA methylation equilibrium. *ROS1* encodes a DNA demethylase, an enzyme that removes DNA methylation catalytically. The ROS1 enzyme functions genome-wide and counteracts DNA methylation pathways such as RdDM. In the *ROS1* promoter, MEMS (DNA methylation monitoring sequence), a sequence adjacent to a *Helitron* TE, is critical for regulating the expression of *ROS1*. Somewhat counter-intuitively, *ROS1* expression is promoted when this sequence is methylated (e.g., by RdDM) and inhibited by demethylation (e.g., by ROS1 itself). Upon expression, the ROS1 enzyme demethylates its own promoter thus reducing its own expression. Reduced ROS1 activity allows then for increased *ROS1* promoter methylation and expression until an equilibrium is reached (Sigman and Slotkin, 2016).

Increase in *BONSAI* Methylation 1 encodes a histone demethylase which is involved in preventing spread of DNA methylation into genes and regulating genome-wide DNA methylation patterns via a feedback loop. *IBM1* contains a heterochromatic repeat in one of its introns. Reduced DNA methylation of this repeat element (e.g., in a mutant background) results in reduced IBM1 expression (improper polyadenylation), followed by increased DNA methylation (mCHG) (Lei et al., 2014; Sigman and Slotkin, 2016; Zhang et al., 2018).

While the exact mechanisms described here for examples in *Arabidopsis* are likely species-specific, similar mechanisms may exist in other plants. The expansion of epigenetic studies from *Arabidopsis* to other systems will be essential to understand which mechanisms are evolutionarily conserved and which are species specific.

## DNA Methylation of the Gene Body

Methylation of actively transcribed genes (predominantly exons: gene body methylation, GbM) is another feature occurring in plant genomes. Although DNA methylation at promoters and transcriptional start sites (gene promoter methylation) has been associated with transcriptional repression, GbM does not generally repress gene expression. Instead, GbM genes are typically expressed constitutively in a wide range of tissues and conditions at moderate levels (housekeeping genes) (Zemach et al., 2010; Bewick et al., 2017). Body-methylated genes thus represent a distinct set of genes, and comprise, for example, approximately 18% of the genes in *Arabidopsis thaliana* ecotype Columbia (Col-0) (Takuno and Gaut, 2012).

Whereas methylation of TEs, and methylation of gene control regions, are apparently ancient in plants, it appears that GbM has expanded in plants more recently, as GbM appears to be minimal in early diverging lineages of land plants (Takuno et al., 2016). However, recent work (Schmid et al., 2018) examining various stages of the *Marchantia* life cycle has shown that GbM is not absent from *Marchantia*, merely prominent in particular stages (it is abundant in the antherozoids). A recent study of *Physcomitrella patens* also revealed that ca. 5.7% protein-coding genes have at least one methylated position in their gene body (Lang et al., 2018). In animals, the situation may be rather different: gene body methylation appears to be general and ancient (Dixon et al., 2016).

Despite ongoing work, the function of GbM still remains mysterious. DNA methylation is potentially mutagenic as spontaneous deamination can convert 5-methylcytosine into thymine, thus the retention of GbM likely comes at a cost. Nevertheless, GbM genes share conserved features and their occurrence spans at least 400 Myr of land plant evolution (Zilberman, 2017). Given the conservation of GbM in evolution (Takuno and Gaut, 2013), GbM genes might be expected to play important roles in plant development. This is supported by *Arabidopsis* mutants. Mutants with severely reduced GbM but largely intact TE methylation show a number of morphological and developmental defects, a pattern that is even observed over progressive generations (Mathieu et al., 2007; Stroud et al., 2013). However, secondary loss of GbM in two Brassicaceae species, *C. planisiliqua* and *E. salsugineum*, indicates that GbM is non-essential over evolutionary time (Bewick et al., 2016).

This paradox, that GbM is likely important but also dispensable, remains to be resolved. Numerous potential functions for GbM have been proposed. These include: (1) involvement in accurate transcription and splicing; (2) the repression of cryptic intragenic promoters (Jeltsch, 2010; Zemach and Zilberman, 2010; Takuno and Gaut, 2012); and (3) sheltering genes from TE insertions (To et al., 2011) while functionless alternatives have also been discussed (Roudier et al., 2009).

## Methylation and the Evolution of Morphology: Examples

### Dioecy

**FIGURE 4:**
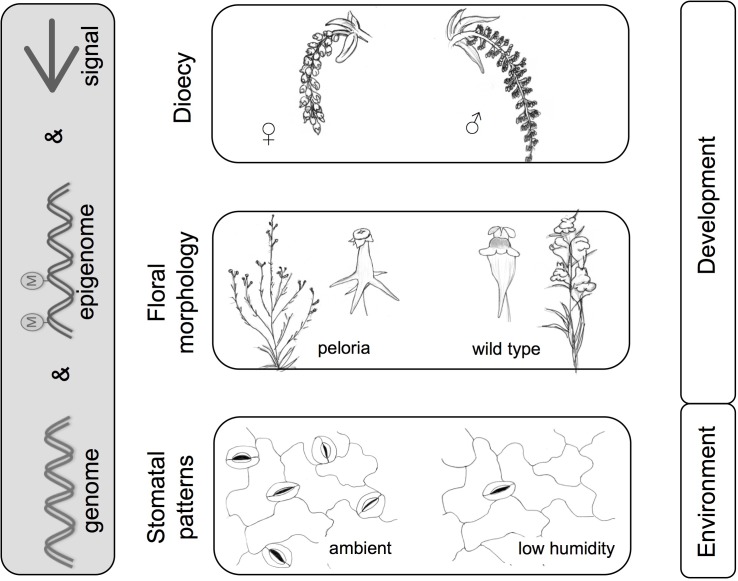
Methylation and morphology – examples from microevolution. Morphological patterns are shaped by the interplay between genome, epigenome, and signals (internal or external). Examples illustrated here include dioecy (poplar catkins); floral morphology in *Linaria vulgaris* (wild type and peloric form), and environmentally influenced stomatal patterns in arabidopsis. Images are adapted from Köhler’s Medicinal Plants and De Vries, 1910. Examples refer to various studies mentioned in text (Cubas et al., 1999; Song et al., 2013a,b; Tricker et al., 2013a,b; Geraldes et al., 2015; Bräutigam et al., 2017).

One of the best supported roles for the action of DNA methylation in plant evolution is provided by the evolution of separate sexes in plants from a cosexual ancestor (**Figure 4**). Dioecy has evolved independently in multiple lineages and it has been suggested that methylation might be a key mechanism (Gorelick, 2003). One genus in which this idea has received support is *Populus*. In the Chinese white poplar (*Populus tomentosa*) sex-specific methylation has been implicated (Song et al., 2013a). More recently, a genomic characterization of the *Populus trichocarpa* sex locus (Geraldes et al., 2015) found a methyltransferase (poplar *MET1* homolog) present at the sex determining region (SDR). It is also of interest that a possible methyltransferase has been noted at the SDR of strawberry (Tennessen et al., 2016), an observation that would merit further investigation. Further support for the involvement of methylation in sex determination in poplar has come from the finding that another gene at the poplar SDR, the poplar homolog of the Arabidopsis Response Regulator 17 (ARR17), is markedly sex-specifically methylated (Bräutigam et al., 2017). Male individuals have generally stronger methylation, including at the putative promoter region.

Evidence is also accruing in other systems. Thus, in the dioecious *Silene latifolia* (white campion), treatments with demethylating agents can alter sex expression in the flowers, converting male flowers to hermaphrodite ones (Janoušek et al., 1996). Other dioecy systems that implicate methylation include persimmons (Akagi et al., 2016; Henry et al., 2018) and papaya (Zhang et al., 2008; Liu et al., 2018). In the latter, CHH-context methylation of *HUA1*, an *AGAMOUS* (*AG*) regulator, is associated with sex reversal in papaya. In the Cucurbitaceae, a family known for its flexible sexual systems (from hermaphroditism and monoecy to dioecy), methylation is also implicated in floral sex determination (Martin et al., 2009; Lai et al., 2017). There is now no doubt that sex-specific methylation is a mechanism that has been employed independently multiple times in the evolution of dioecy.

### Peloria

*Linaria vulgaris* (toadflax) has a remarkable floral mutant, first characterized by Linnaeus, called peloria. This results from the ventralization of flowers, leading to flowers with 5 (ventral) spurs instead of one. It is caused by abolition of function of the dorsal identity gene *CYCLOIDEA*. In now classic work this was shown to result from CYCLOIDEA gene repression by methylation (Cubas et al., 1999; **Figure 4**). Teratomorphs derived in this way can persist by vegetative reproduction (clump formation by root buds), but produce little seed so the mutant is semi-lethal as regards sexual reproduction. The inheritance of this feature was investigated by De Vries in “Die Mutationstheorie” (De Vries, 1910). De Vries divided peloric individuals into two types: (1) hemipelagic in which a mixture of peloric and wild type flowers occurred in an inflorescence, sometimes with intermediates, and (2) fully peloric, in which all flowers in the inflorescence are peloric. As hemipelagic plants have some wild type flowers, they are fertile and this likely explains the wide persistence of the potential for abnormal methylation within the species. The epiallele is heritable, although largely recessive. Fully peloric plants crossed with wild-type produce mostly wild-type with a low frequency of hemipelorics (Cubas et al., 1999).

*Linaria vulgaris* therefore seems (as De Vries puts it) to have “an inherited semi-latent character, which manifests itself from time to time” (De Vries, 1910). Given that similar phenotypes are also present in *Linaria purpurea* (Rudall and Bateman, 2003) it may be that the latent character is phylogenetically conserved in the genus and thus a “latent homology” (Cronk, 2002). Currently unexplained are the pleiotropic effects of CYC methylation on other aspects of morphology besides floral ventralization. Fully peloric plants often have a strongly branched inflorescence as opposed to the simple or near simple raceme of the wild type. There are also reported abnormalities of the pollen and capsule (De Vries, 1910). Even though peloria of *Linaria* is a well-studied epimutation, it is far from giving up all its secrets.

Evolutionarily, this epimutation might seem to be a dead-end. However, its persistence, perhaps through the transmission of weak epialleles via hemipelagic forms, potentially allows further mutations (for instance in corolla shape) to be occasionally expressed in a peloric or hemipelagic background. It is not hard to conceive that this could lead to an alternative pollination niche, reduction in infertility and eventually to speciation. In such a case, the “soft” mutation provided by methylation will have been crucial. Eventually the epigenetic basis could be replaced by genetic loss of function mutation, in which case the initial involvement of methylation in the evolution of a new species will be hard if not impossible to discern. A genetic loss of function mutation is less promising as a starting point, as it is likely to be lethal and to be quickly purged from the population. It is worth noting that new lineages have indeed formed from peloria-type changes. An example of this is *Cadia* (Citerne et al., 2006) a peloric legume (although here the peloria is due to dorsalization rather than ventralization and there is no evidence of methylation being involved).

## The Baldwin Effect – a Theoretical Model

**FIGURE 5:**
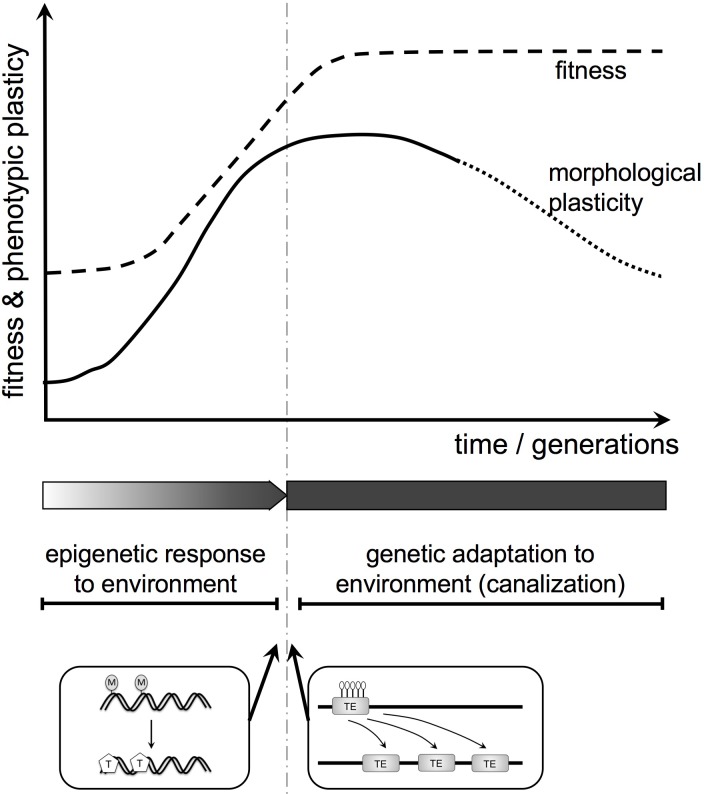
Baldwin effect in plant (morphological) evolution. Here, we integrate the epigenome, phenotypic plasticity, and genetic assimilation into a theoretical evolutionary framework for plant morphology and function. First, environmentally induced changes in the epigenome influence morphology and function. If modified traits increase fitness they will spread. Subsequently, such plastic modifications can be replaced by genetic change. Two examples for potential molecular mechanisms resulting in a transition from epigenetic to genetic change are shown below the diagram. They include the generation of SNPs by the spontaneous deamination of 5-methylcytosine (M) to thymine (T) and transposon activation upon loss of heterochromatic marks (DNA methylation, histone modification; here white lollipops indicate de-methylation).

There is a possible role for environmentally regulated epigenetic control (including methylation) in plant evolution through the Baldwin effect (in the broad sense, including genetic assimilation (Pigliucci et al., 2006; **Figure 5**). The Baldwin effect is usually associated with animals: behavior, being plastic, can change as a learning response to environmental cues, allowing colonization of, and adaptation to, a new environment. Any mutation that gives a genetic predisposition to the changed behavior may be favored as it reduces the cost of learning and increases adaptation to the new environment. Thus, learned behavior can become instinctive behavior.

The same mechanism can apply in plants but here the plastic response is morphological not behavioral. Many complex animals (for instance most vertebrates) show remarkably little morphological phenotypic plasticity. Their plasticity tends to reside in the “extended phenotype,” for instance in behavior. In contrast, the same plant genotype in different environments can look dramatically different, differences that may be stabilized epigenetically. Thus, the Baldwin effect, while a driver of behavioral evolution in animals, may be a driver of morphological evolution in plants. It is a potential mechanism for replacing a phenotypic and epigenetic response to environment with a genetic adaptation to environment (**Figure 5**). These considerations are currently speculative but provide a conceptual framework for future experimental work.

## Concluding Remarks – the Methylation Toolbox and its Application

If, as seems likely, the regulation of genes by methylation evolved from the universal eukaryotic feature of TE defense, then this is an evolutionary change with considerable implications for the evolution of embryophytes. The transition of DNA methylation function from regulation of TEs to regulation of genes, is one of the great evolutionary transitions in the evolution of complex plant life on earth. Methylation of DNA now supplies an important toolbox for fine tuning development, especially when considering its interaction with other epigenetic mechanisms: histone methylation and small RNAs.

However, because of the inherent lability and reversibility of methylation, it may be an “evolutionary sandbox” for the soft exploration of developmental space (Cronk, 2001). It allows for added phenotypic plasticity and infraspecific diversity in the expression of plant morphology (Bossdorf et al., 2008; Bräutigam et al., 2013). Later in evolution, methylation-controlled traits could become hardwired through direct sequence changes, aided by the fact that methylated DNA mutates at a higher rate.

Hypermethylation-based epimutations of a gene, as in peloric *Linaria*, could function as gene knock-downs to allow adaptation prior to gene knock-out and loss. If this is true, then it may be hard to assess the importance of methylation in evolution, as methylation might have been involved in the early stages of the evolution of a number of important traits, but might not be evident now.

Another evolutionarily significant difference between mutation and epimutation lies in exposure to selection (Cronk, 2001; van der Graaf et al., 2015; Vidalis et al., 2016). A conventional loss-of-function recessive mutation will tend to be very rare in a population and in an outbreeding population will be unlikely to occur as the double recessive necessary to generate a selectable phenotype. An epimutation, however, may possibly affect both alleles simultaneously and thus be immediately exposed to selection. There are many elegant studies of the effect of selection on naturally occurring gene mutations, but, with some notable exceptions, e.g., in rice (Zheng et al., 2017), epimutations have been comparatively neglected. This is a challenge for the future.

## Author Contributions

Both authors wrote the paper, made a substantial intellectual contribution to the work, and approved it for publication.

## Conflict of Interest Statement

The authors declare that the research was conducted in the absence of any commercial or financial relationships that could be construed as a potential conflict of interest.
